# Supplementary material for: Social grooming efficiency and techniques are influenced by manual impairment in free-ranging Japanese macaques (Macaca fuscata)
Source: PLoS One. 2020 Feb 21;15(2):e0228978. doi: 10.1371/journal.pone.0228978 (PMC7034802; doi:10.1371/journal.pone.0228978)
Supplement: S1 File — (PDF) [file pone.0228978.s001.pdf]

Table A. Focal Animals Names and Codes

Name	Code	Group	Index of Manual Disability	Age	Kinship (Mother)	Matriline
**Bibi**	BB	Nondisabled	0	12	--	F
**Bow**	BO	Nondisabled	0	6	Bibi	F
**Doro**	DO	Nondisabled	0	6	Erika	J
**Erika**	EK	Nondisabled	0	12	--	J
**Fumin**	FU	Disabled	**0.60**	11	--	H
**Kinchan**	KI	Disabled	**0.51**	12	--	J
**Kobato**	KO	Disabled	**0.63**	14	--	I
**Misaki**	MS	Nondisabled	0	12	--	J
**Miu**	MI	Nondisabled	0	24	--	A
**Momonga**	MG	Nondisabled	0	11	--	E
**Monday**	MO	Nondisabled	0	14	--	B
**Nachan**	NA	Disabled	**0.24**	12	--	D
**Nakami**	NK	Nondisabled	0	8	Kobato	I
**Nanako**	NN	Nondisabled	0	18	--	D
**Natané**	NT	Nondisabled	0	20	--	D
**Pikaru**	PC	Disabled	**0.65**	15	--	I
**Pikoko**	PK	Nondisabled	0	9	Pikaru	I
**Punch98**	PU	Disabled	**0.65**	9	--	J
**Ran**	RA	Disabled	**0.30**	16	--	E
**Ran's daughter**	RD	Nondisabled	0	6	Ran	E
**Ribbon**	RI	Disabled	**0.85**	6	--	B
**Sakurako**	SA	Nondisabled	0	11	--	G
**Sunday**	SU	Nondisabled	0	14	--	B
**Teruko**	TE	Nondisabled	0	12	--	C
**Wendy**	WN	Disabled	**0.25**	8	--	B
**Yokam**	YO	Disabled	**0.18**	6	Yuki	J
**Yuki**	YU	Disabled	**0.76**	21	--	J

Table B. Grooming behavioural ethogram

Behaviour / Movement	Stage	Key code	Code	Description
**Elbow push**	Find egg	Q	ep	Use of the elbow for parting the groomee's hair to find louse eggs
**Hand/Arm push**		W	hp	Use of hand or end of arm for parting the groomee's hair to find louse eggs
**Finger sweep**		E	fs	Use of finger(s) to look for louse egg in groomee's hair
**Hand/Arm sweep**		R	hs	Use of full hand or end of arm to look for louse egg in groomee's hair
**Two-digit pull**		T	sp	Use of two digits to pull hair/skin to look for louse egg in groomee's hair
**Grab groomee's limb**		Y	gl	Take groomee's limbs (including ears) out of the grooming area
**Two-hand/arm/ pinch**	Grip egg	S	tp	Hands, end of arms, or digits are brought together to do a pinch to remove louse eggs or debris from groomee’s hair
**Two-digit pinch**		D	dp	Use two digits to make a pinch to remove louse egg from groomee's hair
**Thumb/ Digit nail loosening**		F	nl	Use digit's nail to loosen louse egg from groomee's hair
**Second hand support**		G	sh	After pulling out the groomee's hair and having both hands in the air, a pinch is made using the thumb and index fingers to remove the louse egg from it.
**See egg on /hand/end of arm**		H	se	Check if removed louse egg is on fingers/hand
**Mouth directly**	Carry egg to mouth	M	md	Direct use of mouth/tongue for removing louse egg from groomee's hair
**Two hand/arm to mouth**		Z	tm	Hands or the ends of arms are brought together to carry the removed louse egg to the mouth
**Single hand/end of arm to mouth**		X	sm	Hand or end of arm is used to carry removed louse egg to the mouth
**Louse egg mastication**	Eat Removed Egg	C	em	Makes a mastication movement after bringing the removed louse egg to mouth
**Infant related behaviors**	Infant related	I	if	Groom infant, carry infant, Remove infant of grooming area
**Other**	Other	O	ob	Distant look, Scratch, wait for change of groomee's position, scan groomee, autogrooming, travel, out of sight, change of groomee

Appendix 1: Behaviour/Movement Images

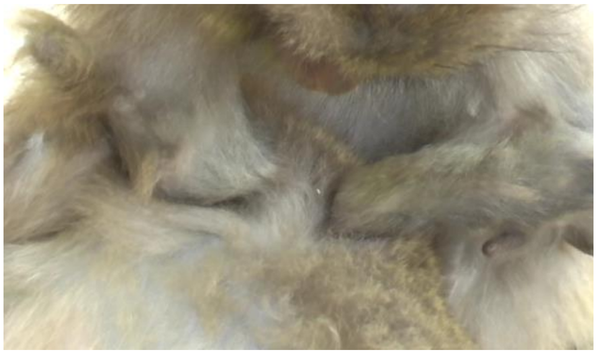

Figure A. Elbow push (ep)

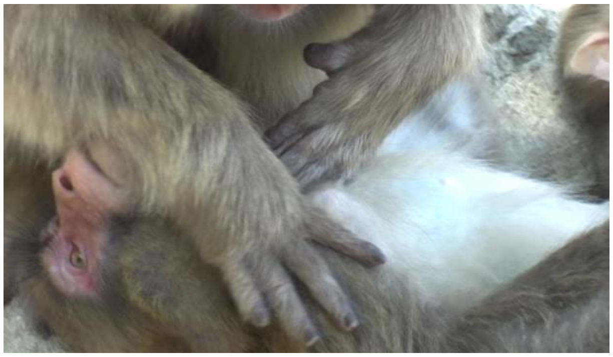

Figure B. Hand/Arm push (hp)

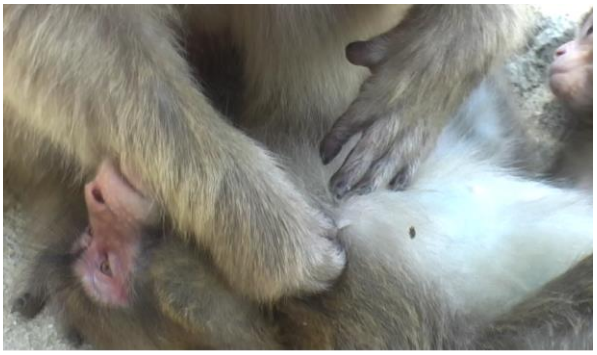

Figure C. Finger sweep (fs)

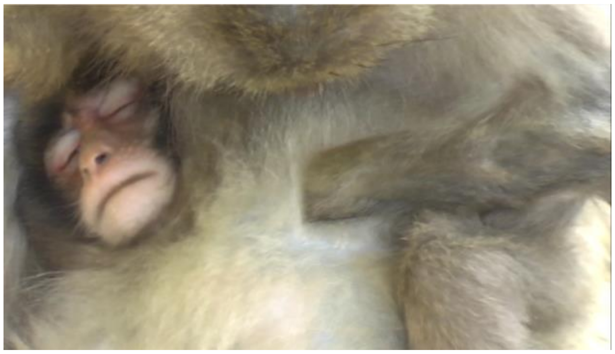

Figure D. Hand/Arm sweep (hs)

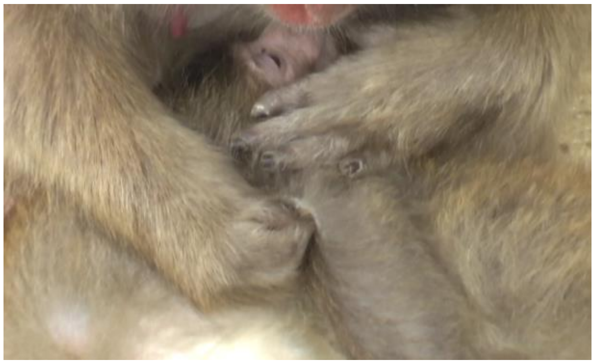

Figure E. Two-digit pull (sp)

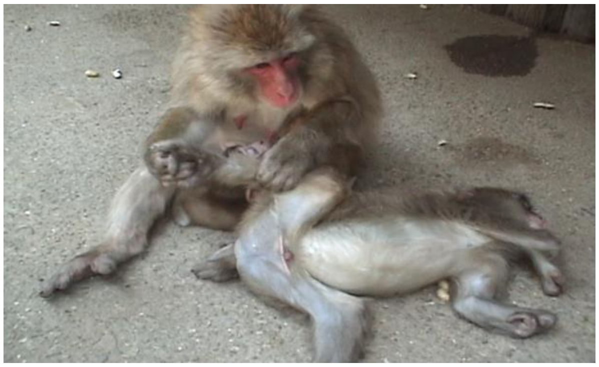

Figure F. Grab groomee's limb (gl)

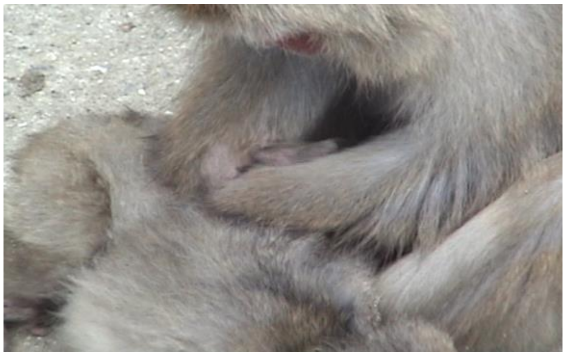

Figure G. Two-hand/arm/digit pinch (tp)

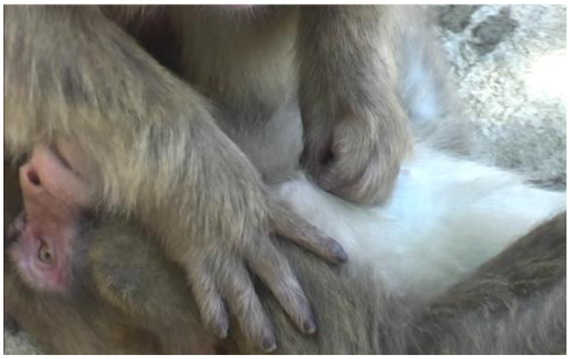

Figure H. Two-digit pinch (dp)

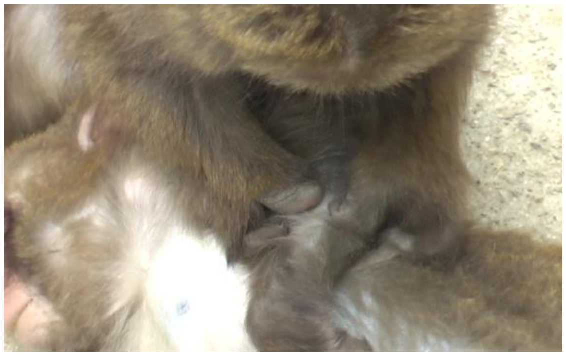

Figure I. Thumb/Digit nail loosening (nl)

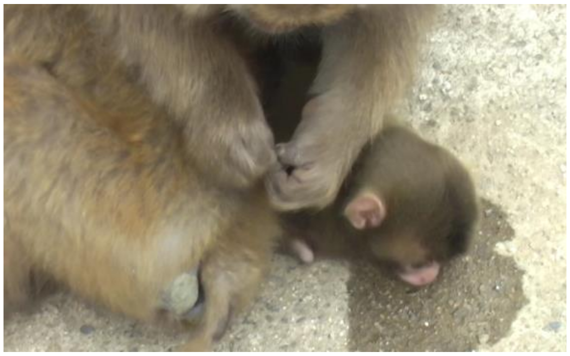

Figure J. Second hand support (sh)

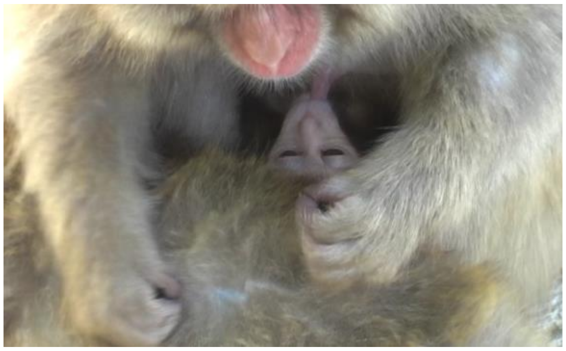

Figure K. See egg in hand (se)

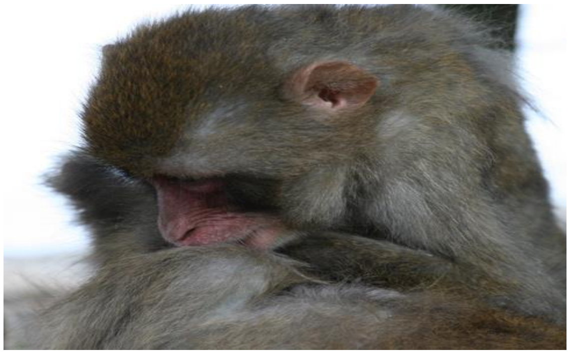

Figure L. Mouth directly (md)

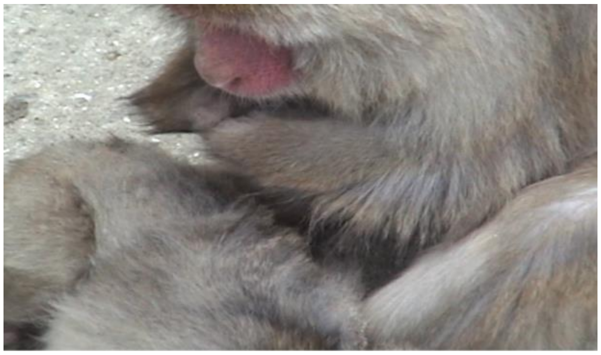

Figure M. Two-hands/arms to mouth (tm)

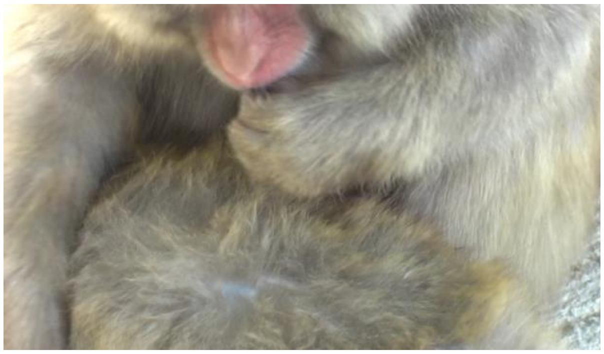

Figure N. Single hand/end of arm to mouth (sm)

Table C. Generalized Mixed-effects models run in this study

Model	Fixed Factor	Random Factor	Observation
1a	Categorical Disability	Individual focal monkey	Number of eggs removed in 2 minutes
1b	Index of Manual Disability	Individual focal monkey	Number of eggs removed in 2 minutes
2a	Categorical Disability	Individual focal monkey	Number of movements performed in 2 minutes
2b	Index of Manual Disability	Individual focal monkey	Number of movements performed in 2 minutes
3a	Categorical Disability	Individual focal monkey	Number of movements performed per egg removed
3b	Index of Manual Disability	Individual focal monkey	Number of movements performed per egg removed
