# Supplementary material for: Social grooming efficiency and techniques are influenced by manual impairment in free-ranging Japanese macaques (Macaca fuscata)
Source: PLoS One. 2020 Feb 21;15(2):e0228978. doi: 10.1371/journal.pone.0228978 (PMC7034802; doi:10.1371/journal.pone.0228978)
Supplement: S2 File — (PDF) [file pone.0228978.s002.pdf]

Table A. Results of generalized linear mixed-effects models (GLMM) examining the relationship between Disability and Index of manual disability with Removal efficiency and Number of movements per 2-minutes

	N	Family	Effects	Factors	Est.	Std. error	z value	Pr(>|z|)
Removal efficiency (number of eggs per 2 mins)	216	Poison	Index of manual disability	Intercept	1.187	0.081	14.69	**< 2e-16**
				Index	-0.602	0.089	-6.77	**1.29e-11**
			Disability	Intercept	0.836	0.166	5.04	**4.78e-07**
				Disabledn	0.8046	0.213	3.79	**0.000153**
Number of movements per 2 mins	216	Poison	Index of manual disability Index	Intercept	4.972	0.030	163.80	**< 2e-16**
				Index	-0.049	0.085	-0.58	0.563
			Disability	Intercept	4.986	0.038	131.66	**< 2e-16**
				Disabledn	-0.041	0.049	-0.83	0.405

Table B. Results of general linear mixed-effects models (LME) examining the relationship between Disability and Index of Manual disability with Movement efficiency

	N	Family	Effects	Factors	Value	Std. Error	t-value	P(>|t|)
Movement efficiency (number of movements per egg removed)	188	Inverse gaussian	Index of manual disability	Intercept	0.082	0.003	25.02	0e+00
				Index	-0.039	0.009	-4.42	**2e-04**
			Disability	Intercept	0.063	0.004	15.23	0e+00
				Disabledn	0.021	0.006	3.76	**9e-04**

Table C. Results of Mann-Whitney test examining differences in the use of the mouth between Disabled and Nondisabled subjects

	Group	N	Mean	W	p-value
Use of mouth	Disabled	108	4.777778	9879.5	**< 2.2e-16**
	Nondisabled	108	0.1296296		
