# Supplementary material for: Social grooming efficiency and techniques are influenced by manual impairment in free-ranging Japanese macaques (Macaca fuscata)
Source: PLoS One. 2020 Feb 21;15(2):e0228978. doi: 10.1371/journal.pone.0228978 (PMC7034802; doi:10.1371/journal.pone.0228978)
Supplement: S3 File — (PDF) [file pone.0228978.s003.pdf]

Table A. Joint Frequencies matrix for ND and NDM grooming techniques

	Target:											
**Given:**	**dp**	**em**	**fs**	**gl**	**hp**	**md**	**nl**	**se**	**sh**	**sm**	**sp**	**Totals**
**dp**	1363	0	82	4	117	2	7	647	37	57	78	2394
**em**	39	0	113	8	256	0	3	47	0	10	90	566
**fs**	372	0	4023	48	1424	2	30	8	4	1	565	6477
**gl**	1	0	61	10	12	0	0	0	0	0	9	93
**hp**	133	0	1667	13	280	0	9	0	0	0	84	2186
**md**	0	3	3	0	3	2	0	0	0	0	2	13
**nl**	37	0	6	0	11	0	47	1	0	0	7	109
**se**	93	57	22	0	34	0	2	6	125	604	37	980
**sh**	6	0	2	0	0	0	0	138	121	30	0	297
**sm**	30	508	11	0	3	0	2	137	8	14	3	716
**sp**	329	0	476	10	44	7	8	1	2	0	411	1288
**Totals**	2403	568	6466	93	2184	13	108	985	297	716	1286	15119

Table B. Transition Frequencies matrix (lag 1) for ND and NDM grooming techniques with probability higher than 15%

	**dp**	**em**	**fs**	**gl**	**hp**	**md**	**nl**	**se**	**sh**	**sm**	**sp**
**dp**	**0.57**	0.00	0.00	0.00	0.00	0.00	0.00	**0.27**	0.00	0.00	0.00
**em**	0.00	0.00	**0.20**	0.00	**0.45**	0.00	0.00	0.00	0.00	0.00	**0.16**
**fs**	0.00	0.00	**0.62**	0.00	**0.22**	0.00	0.00	0.00	0.00	0.00	0.00
**gl**	0.00	0.00	**0.66**	0.00	0.00	0.00	0.00	0.00	0.00	0.00	0.00
**hp**	0.00	0.00	**0.76**	0.00	0.00	0.00	0.00	0.00	0.00	0.00	0.00
**md**	0.00	**0.23**	**0.23**	0.00	**0.23**	**0.15**	0.00	0.00	0.00	0.00	**0.15**
**nl**	**0.34**	0.00	0.00	0.00	0.00	0.00	**0.43**	0.00	0.00	0.00	0.00
**se**	0.00	0.00	0.00	0.00	0.00	0.00	0.00	0.00	0.00	**0.62**	0.00
**sh**	0.00	0.00	0.00	0.00	0.00	0.00	0.00	**0.46**	**0.41**	0.00	0.00
**sm**	0.00	**0.71**	0.00	0.00	0.00	0.00	0.00	**0.19**	0.00	0.00	0.00
**sp**	**0.26**	0.00	**0.37**	0.00	0.00	0.00	0.00	0.00	0.00	0.00	**0.32**

Table C. Transition Frequencies matrix (lag -1) for ND and NDM grooming techniques with probability higher than 15%

	dp	em	fs	gl	hp	md	nl	se	sh	sm	sp
**dp**	**0.57**	0.00	0.00	0.00	0.00	**0.15**	0.00	**0.66**	0.00	0.00	0.00
**em**	0.00	0.00	0.00	0.00	0.00	0.00	0.00	0.00	0.00	0.00	0.00
**fs**	**0.15**	0.00	**0.62**	**0.52**	**0.65**	**0.15**	**0.28**	0.00	0.00	0.00	**0.44**
**gl**	0.00	0.00	0.00	0.00	0.00	0.00	0.00	0.00	0.00	0.00	0.00
**hp**	0.00	0.00	**0.26**	0.00	0.00	0.00	0.00	0.00	0.00	0.00	0.00
**md**	0.00	0.00	0.00	0.00	0.00	**0.15**	0.00	0.00	0.00	0.00	0.00
**nl**	0.00	0.00	0.00	0.00	0.00	0.00	**0.44**	0.00	0.00	0.00	0.00
**se**	0.00	0.00	0.00	0.00	0.00	0.00	0.00	0.00	**0.42**	**0.84**	0.00
**sh**	0.00	0.00	0.00	0.00	0.00	0.00	0.00	0.00	**0.41**	0.00	0.00
**sm**	0.00	**0.89**	0.00	0.00	0.00	0.00	0.00	0.00	0.00	0.00	0.00
**sp**	0.00	0.00	0.00	0.00	0.00	**0.54**	0.00	0.00	0.00	0.00	**0.32**

Table D. Final Transition Frequencies matrix for ND and NDM grooming techniques. There are 32 relevant transitions. Bold values are higher than the average (50%)

	dp	em	fs	gl	hp	md	nl	se	sh	sm	sp
**dp**	**1.14**	0.00	0.00	0.00	0.00	0.15	0.00	**0.93**	0.00	0.00	0.00
**em**	0.00	0.00	0.20	0.00	0.45	0.00	0.00	0.00	0.00	0.00	0.16
**fs**	0.15	0.00	**1.24**	0.52	**0.87**	0.15	0.28	0.00	0.00	0.00	0.44
**gl**	0.00	0.00	**0.66**	0.00	0.00	0.00	0.00	0.00	0.00	0.00	0.00
**hp**	0.00	0.00	**1.02**	0.00	0.00	0.00	0.00	0.00	0.00	0.00	0.00
**md**	0.00	0.23	0.23	0.00	0.23	0.31	0.00	0.00	0.00	0.00	0.15
**nl**	0.34	0.00	0.00	0.00	0.00	0.00	**0.87**	0.00	0.00	0.00	0.00
**se**	0.00	0.00	0.00	0.00	0.00	0.00	0.00	0.00	0.42	**1.46**	0.00
**sh**	0.00	0.00	0.00	0.00	0.00	0.00	0.00	0.46	**0.81**	0.00	0.00
**sm**	0.00	**1.60**	0.00	0.00	0.00	0.00	0.00	0.19	0.00	0.00	0.00
**sp**	0.26	0.00	0.37	0.00	0.00	0.54	0.00	0.00	0.00	0.00	**0.64**

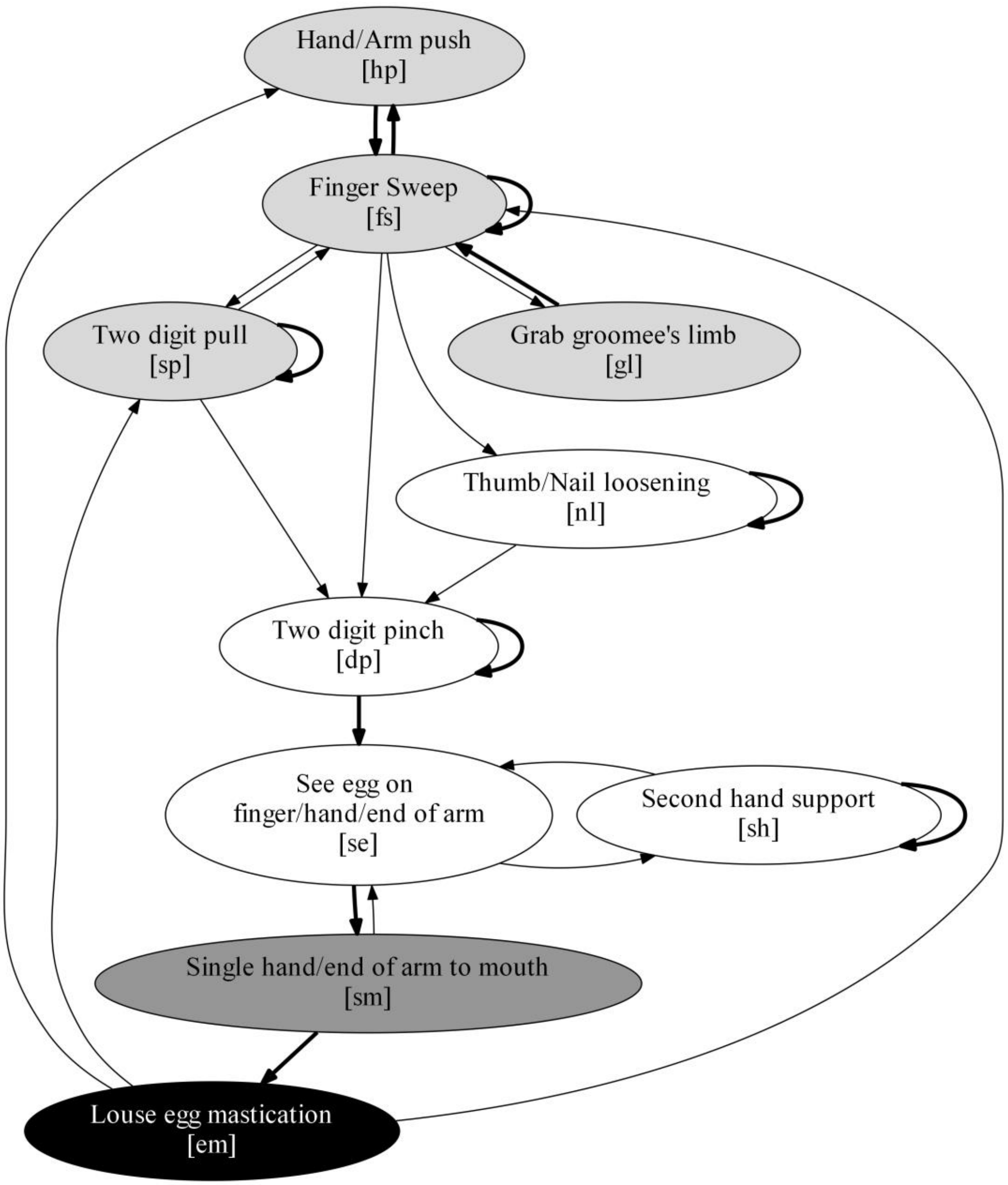

Figure A. Main nondisabled grooming technique (ND)

The process begins at the top of the figure. Node color matches with the four grooming stages described in Fig 4. Lines represent relevant transitions. Thicker lines represent transitions with probabilities higher than 50%.

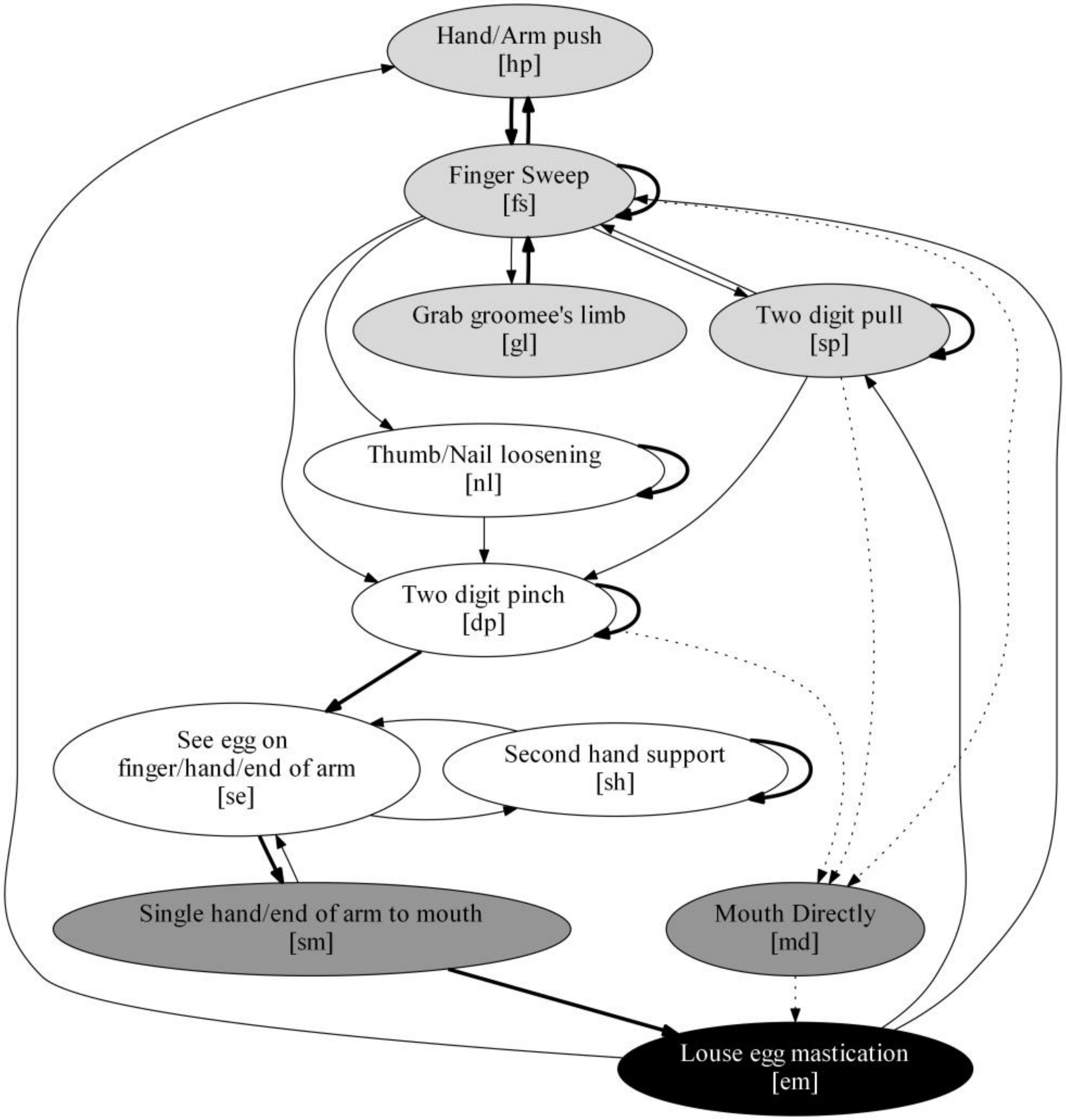

Figure B. Nondisabled grooming technique including the use of the mouth (NDM)

The process begins at the top of the figure. Node color matches with the four grooming stages described in Fig 4. Lines represent relevant transitions. Thicker lines represent transitions with probabilities higher than 50%. Dotted lines show transitions to movements not found in the ND technique.

Table E. Joint Frequencies matrix for DA grooming technique

Given:	Target: dp	em	fs	gl	hp	md	nl	se	sh	sm	sp	Totals
**dp**	387	0	23	0	26	7	14	178	0	8	17	660
**em**	14	0	33	2	83	0	0	12	0	3	2	149
**fs**	117	0	1762	11	654	18	63	4	0	0	81	2710
**gl**	0	0	12	1	8	0	0	0	0	0	0	21
**hp**	26	0	770	4	80	2	8	0	0	0	16	906
**md**	5	11	11	0	11	5	5	0	0	0	1	49
**nl**	35	0	18	1	23	11	105	1	0	0	6	200
**se**	23	29	19	2	9	0	0	2	21	164	3	272
**sh**	0	0	0	0	1	0	0	20	22	6	0	49
**sm**	6	110	6	0	3	0	0	52	5	12	0	194
**sp**	51	0	52	0	6	7	6	3	1	0	41	167
**Totals**	664	150	2706	21	904	50	201	272	49	193	167	5377

Table F. Transition Frequencies matrix (lag 1) for DA grooming technique with probability higher than 15%

	dp	em	fs	gl	hp	md	nl	se	sh	sm	sp
**dp**	**0.59**	0.00	0.00	0.00	0.00	0.00	0.00	**0.27**	0.00	0.00	0.00
**em**	0.00	0.00	**0.22**	0.00	**0.56**	0.00	0.00	0.00	0.00	0.00	0.00
**fs**	0.00	0.00	**0.65**	0.00	**0.24**	0.00	0.00	0.00	0.00	0.00	0.00
**gl**	0.00	0.00	**0.57**	0.00	**0.38**	0.00	0.00	0.00	0.00	0.00	0.00
**hp**	0.00	0.00	**0.85**	0.00	0.00	0.00	0.00	0.00	0.00	0.00	0.00
**md**	0.00	**0.22**	**0.22**	0.00	**0.22**	0.00	0.00	0.00	0.00	0.00	0.00
**nl**	**0.18**	0.00	0.00	0.00	0.00	0.00	**0.53**	0.00	0.00	0.00	0.00
**se**	0.00	0.00	0.00	0.00	0.00	0.00	0.00	0.00	0.00	0.60	0.00
**sh**	0.00	0.00	0.00	0.00	0.00	0.00	0.00	**0.41**	**0.45**	0.00	0.00
**sm**	0.00	**0.57**	0.00	0.00	0.00	0.00	0.00	**0.27**	0.00	0.00	0.00
**sp**	**0.31**	0.00	**0.31**	0.00	0.00	0.00	0.00	0.00	0.00	0.00	**0.25**

Table G. Transition Frequencies matrix (lag -1) for DA grooming technique with probability higher than 15%

Given:	dp	em	fs	gl	hp	md	nl	se	sh	sm	sp
**dp**	**0.58**	0.00	0.00	0.00	0.00	0.00	0.00	0.65	0.00	0.00	0.00
**em**	0.00	0.00	0.00	0.00	0.00	0.00	0.00	0.00	0.00	0.00	0.00
**fs**	**0.18**	0.00	**0.65**	**0.52**	**0.72**	**0.36**	**0.31**	0.00	0.00	0.00	**0.49**
**gl**	0.00	0.00	0.00	0.00	0.00	0.00	0.00	0.00	0.00	0.00	0.00
**hp**	0.00	0.00	**0.28**	**0.19**	0.00	0.00	0.00	0.00	0.00	0.00	0.00
**md**	0.00	0.00	0.00	0.00	0.00	0.00	0.00	0.00	0.00	0.00	0.00
**nl**	0.00	0.00	0.00	0.00	0.00	**0.22**	**0.52**	0.00	0.00	0.00	0.00
**se**	0.00	**0.19**	0.00	0.00	0.00	0.00	0.00	0.00	**0.43**	**0.85**	0.00
**sh**	0.00	0.00	0.00	0.00	0.00	0.00	0.00	0.00	**0.45**	0.00	0.00
**sm**	0.00	**0.73**	0.00	0.00	0.00	0.00	0.00	**0.19**	0.00	0.00	0.00
**sp**	0.00	0.00	0.00	0.00	0.00	0.00	0.00	0.00	0.00	0.00	**0.25**

Table H. Final Transition Frequencies matrix for DA grooming technique. There are 31 relevant transitions. Bold values are higher than the average (57%)

	dp	em	fs	gl	hp	md	nl	se	sh	sm	sp
**dp**	**1.17**	0.00	0.00	0.00	0.00	0.00	0.00	**0.92**	0.00	0.00	0.00
**em**	0.00	0.00	0.22	0.00	0.56	0.00	0.00	0.00	0.00	0.00	0.00
**fs**	0.18	0.00	**1.30**	0.52	**0.96**	0.36	0.31	0.00	0.00	0.00	0.49
**gl**	0.00	0.00	**0.57**	0.00	0.38	0.00	0.00	0.00	0.00	0.00	0.00
**hp**	0.00	0.00	**1.13**	0.19	0.00	0.00	0.00	0.00	0.00	0.00	0.00
**md**	0.00	0.22	0.22	0.00	0.22	0.00	0.00	0.00	0.00	0.00	0.00
**nl**	0.18	0.00	0.00	0.00	0.00	0.22	**1.05**	0.00	0.00	0.00	0.00
**se**	0.00	0.19	0.00	0.00	0.00	0.00	0.00	0.00	0.43	**1.45**	0.00
**sh**	0.00	0.00	0.00	0.00	0.00	0.00	0.00	0.41	**0.90**	0.00	0.00
**sm**	0.00	**1.30**	0.00	0.00	0.00	0.00	0.00	0.46	0.00	0.00	0.00
**sp**	0.31	0.00	0.31	0.00	0.00	0.00	0.00	0.00	0.00	0.00	0.49

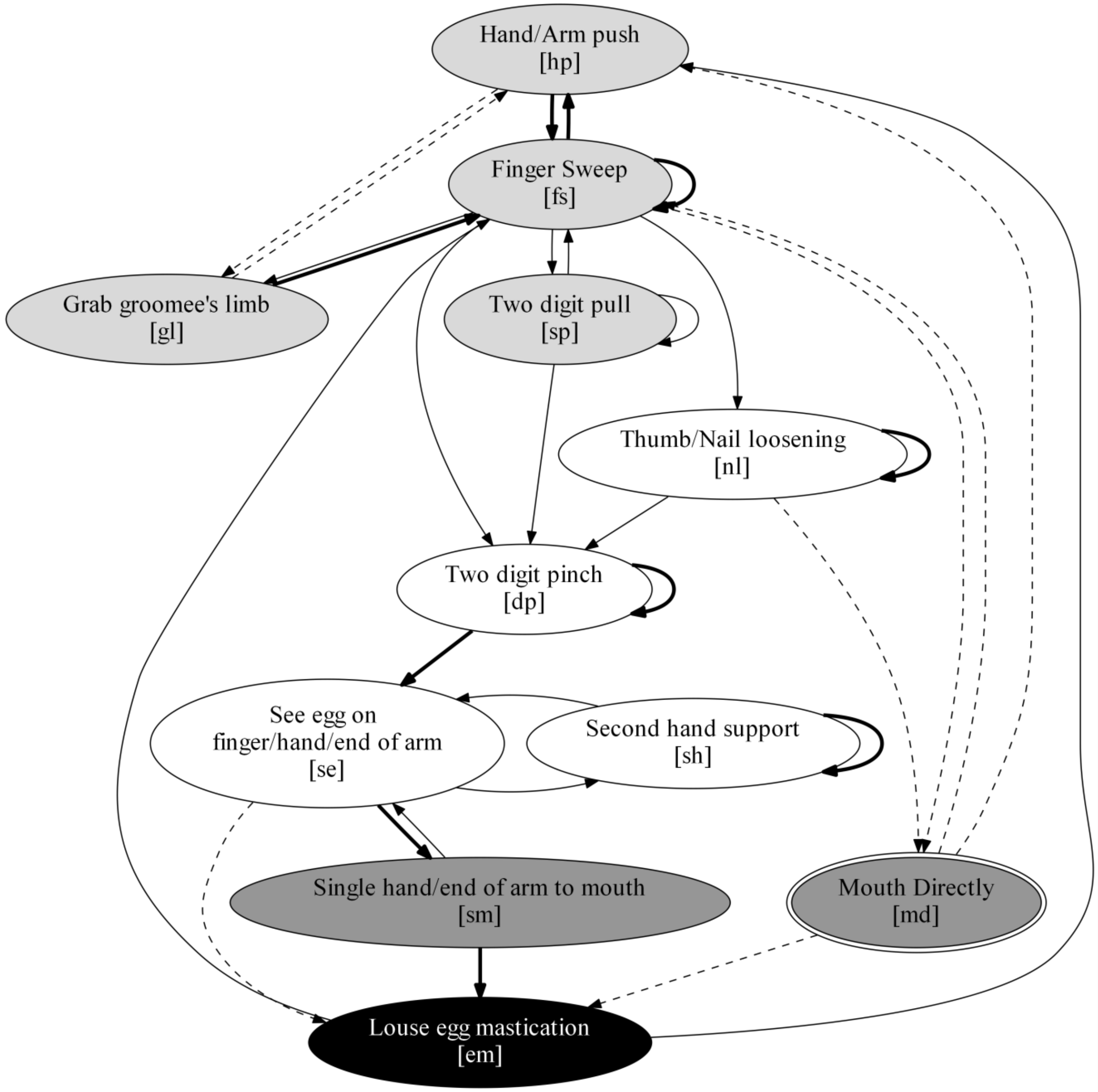

Figure C. Disabled grooming technique DA

The process begins at the top of the figure. Node color matches with the four grooming stages described in Fig 4. Solid lines represent transitions common with the ND technique. Dashed lines show transitions not found in the ND technique. Transitions with probabilities higher than 57% are indicated with thicker lines. Double-bordered movements (1) are performed only by the disabled individuals that use this technique.

Table I. Joint Frequencies matrix for DB grooming technique

	**Target:**												
**Given:**	**dp**	**em**	**fs**	**gl**	**hp**	**md**	**nl**	**se**	**sh**	**sm**	**sp**	**tm**	**Totals**
**dp**	44	0	9	0	4	4	4	20	0	6	1	0	92
**em**	0	0	12	0	33	0	0	2	0	0	0	0	47
**fs**	21	0	827	3	319	17	45	0	0	2	23	0	1257
**gl**	0	0	4	0	1	0	0	0	0	0	0	0	5
**hp**	3	0	352	2	31	2	17	0	0	0	3	0	410
**md**	4	23	14	0	2	14	2	0	0	0	3	0	62
**nl**	16	0	18	0	14	18	123	1	0	1	3	0	194
**se**	2	6	2	1	1	0	1	0	2	18	0	0	33
**sh**	0	0	0	0	0	0	0	0	1	1	0	1	3
**sm**	0	18	1	0	0	0	0	9	0	0	0	0	28
**sp**	1	0	18	0	3	7	4	0	0	0	6	0	39
**tm**	0	0	0	0	0	0	0	1	0	0	0	0	1
**Totals**	91	47	1257	6	408	62	196	33	3	28	39	1	2171

Table J. Transition Frequencies matrix (lag 1) for DB grooming technique with probability higher than 15%

	**dp**	**em**	**fs**	**gl**	**hp**	**md**	**nl**	**se**	**sh**	**sm**	**sp**	**tm**
**dp**	**0.48**	0.00	0.00	0.00	0.00	0.00	0.00	**0.22**	0.00	0.00	0.00	0.00
**em**	0.00	0.00	**0.26**	0.00	**0.70**	0.00	0.00	0.00	0.00	0.00	0.00	0.00
**fs**	0.00	0.00	**0.66**	0.00	**0.25**	0.00	0.00	0.00	0.00	0.00	0.00	0.00
**gl**	0.00	0.00	**0.80**	0.00	**0.20**	0.00	0.00	0.00	0.00	0.00	0.00	0.00
**hp**	0.00	0.00	**0.86**	0.00	0.00	0.00	0.00	0.00	0.00	0.00	0.00	0.00
**md**	0.00	**0.37**	**0.23**	0.00	0.00	**0.23**	0.00	0.00	0.00	0.00	0.00	0.00
**nl**	0.00	0.00	0.00	0.00	0.00	0.00	**0.63**	0.00	0.00	0.00	0.00	0.00
**se**	0.00	**0.18**	0.00	0.00	0.00	0.00	0.00	0.00	0.00	**0.55**	0.00	0.00
**sh**	0.00	0.00	0.00	0.00	0.00	0.00	0.00	0.00	**0.33**	**0.33**	0.00	**0.33**
**sm**	0.00	**0.64**	0.00	0.00	0.00	0.00	0.00	**0.32**	0.00	0.00	0.00	0.00
**sp**	0.00	0.00	**0.46**	0.00	0.00	**0.18**	0.00	0.00	0.00	0.00	**0.15**	0.00
**tm**	0.00	0.00	0.00	0.00	0.00	0.00	0.00	**1.00**	0.00	0.00	0.00	0.00

Table K. Transition Frequencies matrix (lag -1) for DB grooming technique with probability higher than 15%

	dp	em	fs	gl	hp	md	nl	se	sh	sm	sp	tm
**dp**	**0.48**	0.00	0.00	0.00	0.00	0.00	0.00	**0.61**	0.00	**0.21**	0.00	0.00
**em**	0.00	0.00	0.00	0.00	0.00	0.00	0.00	0.00	0.00	0.00	0.00	0.00
**fs**	**0.23**	0.00	**0.66**	**0.50**	**0.78**	**0.27**	**0.23**	0.00	0.00	0.00	**0.59**	0.00
**gl**	0.00	0.00	0.00	0.00	0.00	0.00	0.00	0.00	0.00	0.00	0.00	0.00
**hp**	0.00	0.00	**0.28**	**0.33**	0.00	0.00	0.00	0.00	0.00	0.00	0.00	0.00
**md**	0.00	**0.49**	0.00	0.00	0.00	**0.23**	0.00	0.00	0.00	0.00	0.00	0.00
**nl**	**0.18**	0.00	0.00	0.00	0.00	**0.29**	**0.63**	0.00	0.00	0.00	0.00	0.00
**se**	0.00	0.00	0.00	**0.17**	0.00	0.00	0.00	0.00	**0.67**	**0.64**	0.00	0.00
**sh**	0.00	0.00	0.00	0.00	0.00	0.00	0.00	0.00	**0.33**	0.00	0.00	**1.00**
**sm**	0.00	**0.38**	0.00	0.00	0.00	0.00	0.00	**0.27**	0.00	0.00	0.00	0.00
**sp**	0.00	0.00	0.00	0.00	0.00	0.00	0.00	0.00	0.00	0.00	**0.15**	0.00
**tm**	0.00	0.00	0.00	0.00	0.00	0.00	0.00	0.00	0.00	0.00	0.00	0.00

Table L. Final Transition Frequencies matrix for DB grooming technique. There are 35 relevant transitions. Bold values are higher than the average (59%)

	dp	em	fs	gl	hp	md	nl	se	sh	sm	sp	tm
**dp**	**0.96**	0.00	0.00	0.00	0.00	0.00	0.00	**0.82**	0.00	0.21	0.00	0.00
**em**	0.00	0.00	0.26	0.00	**0.70**	0.00	0.00	0.00	0.00	0.00	0.00	0.00
**fs**	0.23	0.00	**1.32**	0.50	**1.04**	0.27	0.23	0.00	0.00	0.00	0.59	0.00
**gl**	0.00	0.00	**0.80**	0.00	0.20	0.00	0.00	0.00	0.00	0.00	0.00	0.00
**hp**	0.00	0.00	**1.14**	0.33	0.00	0.00	0.00	0.00	0.00	0.00	0.00	0.00
**md**	0.00	**0.86**	0.23	0.00	0.00	0.45	0.00	0.00	0.00	0.00	0.00	0.00
**nl**	0.18	0.00	0.00	0.00	0.00	0.29	**1.26**	0.00	0.00	0.00	0.00	0.00
**se**	0.00	0.18	0.00	0.17	0.00	0.00	0.00	0.00	**0.67**	**1.19**	0.00	0.00
**sh**	0.00	0.00	0.00	0.00	0.00	0.00	0.00	0.00	**0.67**	0.33	0.00	**1.33**
**sm**	0.00	**1.03**	0.00	0.00	0.00	0.00	0.00	0.59	0.00	0.00	0.00	0.00
**sp**	0.00	0.00	0.46	0.00	0.00	0.18	0.00	0.00	0.00	0.00	0.31	0.00
**tm**	0.00	0.00	0.00	0.00	0.00	0.00	0.00	**1.00**	0.00	0.00	0.00	0.00

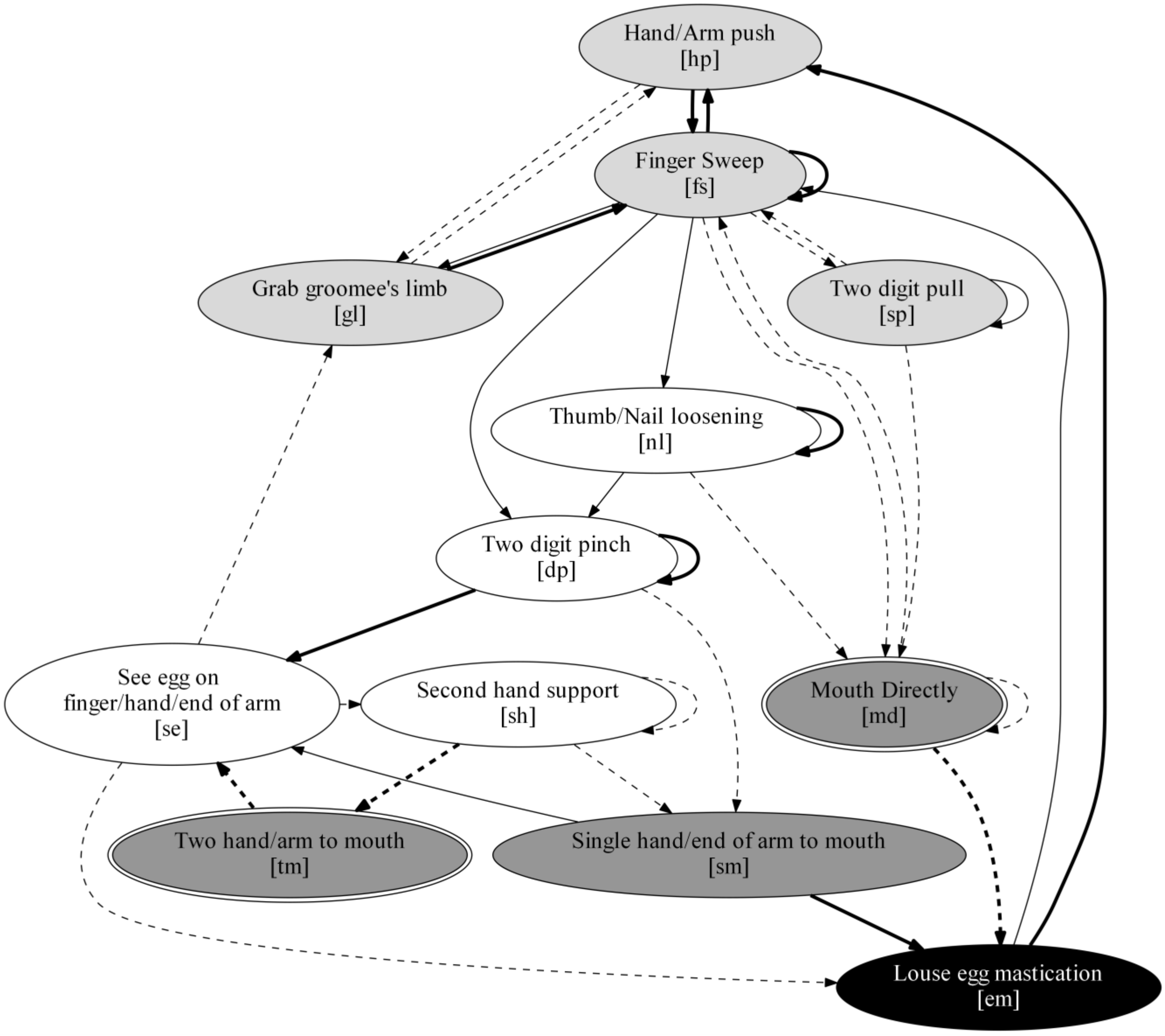

Figure D. Disabled grooming technique DB

The process begins at the top of the figure. Node color matches with the four grooming stages described in Fig 4. Solid lines represent transitions common with the ND technique. Dashed lines show transitions not found in the ND technique. Transitions with probabilities higher than 59% are indicated with thicker lines. Double-bordered movements (2) are performed only by the disabled individuals that use this technique.

Table M. Joint Frequencies matrix for DC grooming technique

Given:	Target: em	fs	gl	hp	md	nl	se	sm	tm	tp	Totals
**em**	0	21	0	22	0	0	1	0	0	0	44
**fs**	0	2200	18	409	111	47	3	2	0	24	2814
**gl**	0	20	0	2	0	0	0	0	0	0	22
**hp**	0	450	1	46	10	5	0	0	0	0	512
**md**	36	75	2	16	12	2	0	0	1	0	144
**nl**	0	30	0	11	11	111	2	0	0	0	165
**se**	5	6	1	3	0	0	1	3	4	0	23
**sm**	2	0	0	0	0	0	3	0	0	0	5
**tm**	1	0	0	0	0	0	4	0	0	0	5
**tp**	0	14	0	1	0	0	9	0	0	1	25
**Totals**	44	2816	22	510	144	165	23	5	5	25	3759

Table N. Transition Frequencies matrix (lag 1) for DC grooming technique with probability higher than 15%

	em	fs	gl	hp	md	nl	se	sm	tm	tp
**em**	0.00	**0.48**	0.00	**0.50**	0.00	0.00	0.00	0.00	0.00	0.00
**fs**	0.00	**0.78**	0.00	0.00	0.00	0.00	0.00	0.00	0.00	0.00
**gl**	0.00	**0.91**	0.00	0.00	0.00	0.00	0.00	0.00	0.00	0.00
**hp**	0.00	**0.88**	0.00	0.00	0.00	0.00	0.00	0.00	0.00	0.00
**md**	**0.25**	**0.52**	0.00	0.00	0.00	0.00	0.00	0.00	0.00	0.00
**nl**	0.00	**0.18**	0.00	0.00	0.00	**0.67**	0.00	0.00	0.00	0.00
**se**	**0.22**	**0.26**	0.00	0.00	0.00	0.00	0.00	0.00	**0.17**	0.00
**sm**	**0.40**	0.00	0.00	0.00	0.00	0.00	**0.60**	0.00	0.00	0.00
**tm**	**0.20**	0.00	0.00	0.00	0.00	0.00	**0.80**	0.00	0.00	0.00
**tp**	0.00	**0.56**	0.00	0.00	0.00	0.00	**0.36**	0.00	0.00	0.00

Table O. Transition Frequencies matrix (lag -1) for DC grooming technique with probability higher than 15%

	em	fs	gl	hp	md	nl	se	sm	tm	tp
**em**	0.00	0.00	0.00	0.00	0.00	0.00	0.00	0.00	0.00	0.00
**fs**	0.00	**0.78**	**0.82**	**0.80**	**0.77**	**0.28**	0.00	**0.40**	0.00	**0.96**
**gl**	0.00	0.00	0.00	0.00	0.00	0.00	0.00	0.00	0.00	0.00
**hp**	0.00	**0.16**	0.00	0.00	0.00	0.00	0.00	0.00	0.00	0.00
**md**	**0.82**	0.00	0.00	0.00	0.00	0.00	0.00	0.00	**0.20**	0.00
**nl**	0.00	0.00	0.00	0.00	0.00	**0.67**	0.00	0.00	0.00	0.00
**se**	0.00	0.00	0.00	0.00	0.00	0.00	0.00	**0.60**	**0.80**	0.00
**sm**	0.00	0.00	0.00	0.00	0.00	0.00	0.00	0.00	0.00	0.00
**tm**	0.00	0.00	0.00	0.00	0.00	0.00	**0.17**	0.00	0.00	0.00
**tp**	0.00	0.00	0.00	0.00	0.00	0.00	**0.39**	0.00	0.00	0.00

Table P. Final Transition Frequencies matrix for DC grooming technique. There are 26 relevant transitions. Bold values are higher than the average (67%)

	em	fs	gl	hp	md	nl	se	sm	tm	tp
**em**	0.00	0.48	0.00	0.50	0.00	0.00	0.00	0.00	0.00	0.00
**fs**	0.00	**1.56**	**0.82**	**0.80**	**0.77**	0.28	0.00	0.40	0.00	**0.96**
**gl**	0.00	**0.91**	0.00	0.00	0.00	0.00	0.00	0.00	0.00	0.00
**hp**	0.00	**1.04**	0.00	0.00	0.00	0.00	0.00	0.00	0.00	0.00
**md**	**1.07**	0.52	0.00	0.00	0.00	0.00	0.00	0.00	0.20	0.00
**nl**	0.00	0.18	0.00	0.00	0.00	**1.35**	0.00	0.00	0.00	0.00
**se**	0.22	0.26	0.00	0.00	0.00	0.00	0.00	0.60	**0.97**	0.00
**sm**	0.40	0.00	0.00	0.00	0.00	0.00	0.60	0.00	0.00	0.00
**tm**	0.20	0.00	0.00	0.00	0.00	0.00	**0.97**	0.00	0.00	0.00
**tp**	0.00	0.56	0.00	0.00	0.00	0.00	**0.75**	0.00	0.00	0.00

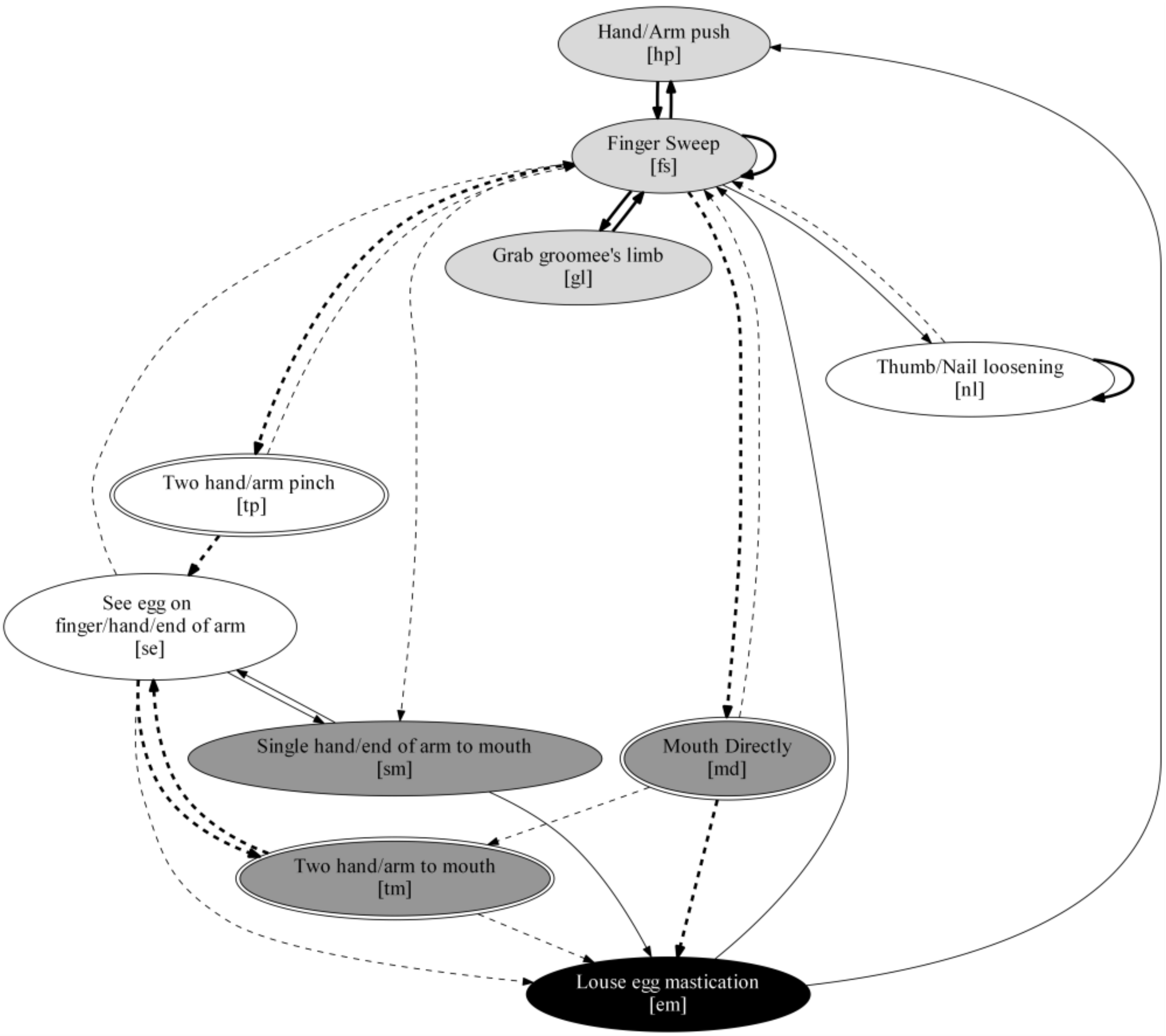

Figure E. Disabled grooming technique DC

The process begins at the top of the figure. Node color matches with the four grooming stages described in Fig 4. Solid lines represent transitions common with the ND technique. Dashed lines show transitions not found in the ND technique. Transitions with probabilities higher than 67% are indicated with thicker lines. Double-bordered movements (3) are performed only by the disabled individuals that use this technique.

Table Q. Joint Frequencies matrix for DD grooming technique

Given:	Target: em	fs	gl	hp	md	nl	Totals
**em**	0	9	1	5	0	0	15
**fs**	1	635	5	200	75	7	923
**gl**	0	5	0	1	0	0	6
**hp**	0	201	0	15	15	1	232
**md**	15	72	0	7	14	0	108
**nl**	0	1	0	4	3	16	24
**Totals**	16	923	6	232	107	24	1308

Table R. Transition Frequencies matrix (lag 1) for DD grooming technique with probability higher than 15%

	em	fs	gl	hp	md	nl
**em**	0.00	**0.60**	0.00	**0.33**	0.00	0.00
**fs**	0.00	**0.69**	0.00	**0.22**	0.00	0.00
**gl**	0.00	**0.83**	0.00	**0.17**	0.00	0.00
**hp**	0.00	**0.87**	0.00	0.00	0.00	0.00
**md**	0.00	**0.67**	0.00	0.00	0.00	0.00
**nl**	0.00	0.00	0.00	**0.17**	0.00	**0.67**

Table S. Transition Frequencies matrix (lag -1) for DD grooming technique with probability higher than 15%

	em	fs	gl	hp	md	nl
**em**	0.00	0.00	**0.17**	0.00	0.00	0.00
**fs**	0.00	**0.69**	**0.83**	**0.86**	**0.70**	**0.29**
**gl**	0.00	0.00	0.00	0.00	0.00	0.00
**hp**	0.00	**0.22**	0.00	0.00	0.00	0.00
**md**	**0.94**	0.00	0.00	0.00	0.00	0.00
**nl**	0.00	0.00	0.00	0.00	0.00	**0.67**

Table T. Final Transition Frequencies matrix for DD grooming technique. There are 15 relevant transitions. Bold values are higher than the average (70%)

	**em**	**fs**	**gl**	**hp**	**md**	**nl**
**em**	0.00	0.60	0.17	0.33	0.00	0.00
**fs**	0.00	**1.38**	**0.83**	**1.08**	0.70	0.29
**gl**	0.00	**0.83**	0.00	0.17	0.00	0.00
**hp**	0.00	**1.08**	0.00	0.00	0.00	0.00
**md**	**0.94**	0.67	0.00	0.00	0.00	0.00
**nl**	0.00	0.00	0.00	0.17	0.00	**1.33**

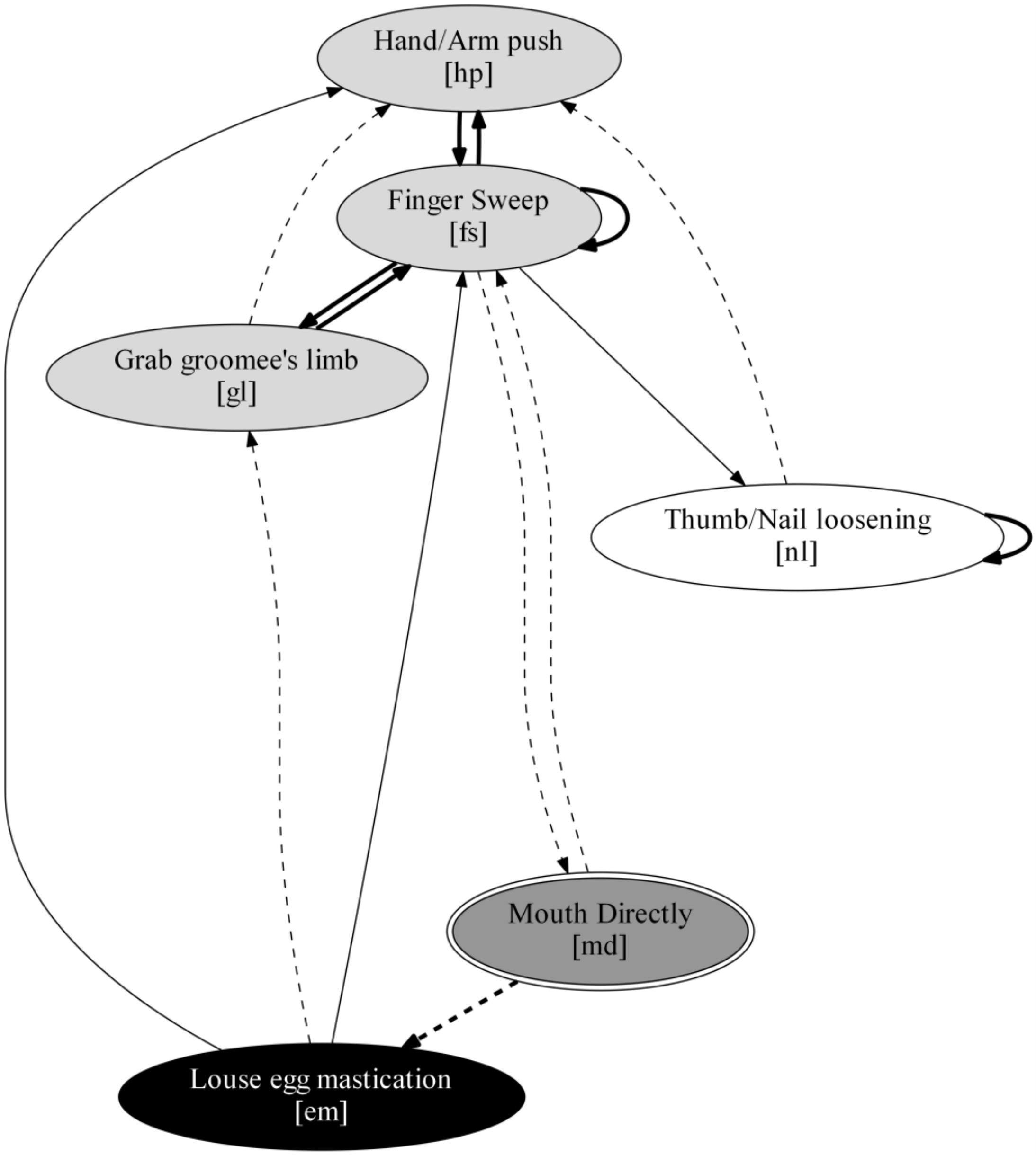

Figure F. Disabled grooming technique DD

The process begins at the top of the figure. Node color matches with the four grooming stages described in Fig 4. Solid lines represent transitions common with the ND technique. Dashed lines show transitions not found in the ND technique. Transitions with probabilities higher than 70% are indicated with thicker lines. Double-bordered movements (1) are performed only by the disabled individuals that use this technique.

Table U. Joint Frequencies matrix for DE grooming technique

Given:	Target: em	fs	gl	hp	hs	md	se	sm	tm	tp	Totals
**em**	0	0	0	4	1	0	2	0	0	0	7
**fs**	0	2	0	2	0	0	0	0	0	1	5
**gl**	0	0	5	0	16	0	0	0	0	0	21
**hp**	0	2	3	28	152	0	0	0	0	9	194
**hs**	0	0	13	152	628	19	0	1	0	73	886
**md**	0	0	0	2	20	0	0	0	0	1	23
**se**	3	0	0	0	3	0	0	0	3	0	9
**sm**	2	0	0	0	0	0	0	0	0	0	2
**tm**	2	2	0	2	1	0	5	0	0	0	12
**tp**	0	0	0	5	62	4	2	1	9	25	108
**Totals**	7	6	21	195	883	23	9	2	12	109	1267

Table V. Transition Frequencies matrix (lag 1) for DE grooming technique with probability higher than 15%

	em	fs	gl	hp	hs	md	se	sm	tm	tp
**em**	0.00	0.00	0.00	**0.57**	0.00	0.00	**0.29**	0.00	0.00	0.00
**fs**	0.00	**0.40**	0.00	**0.40**	0.00	0.00	0.00	0.00	0.00	**0.20**
**gl**	0.00	0.00	**0.24**	0.00	**0.76**	0.00	0.00	0.00	0.00	0.00
**hp**	0.00	0.00	0.00	0.00	**0.78**	0.00	0.00	0.00	0.00	0.00
**hs**	0.00	0.00	0.00	**0.17**	**0.71**	0.00	0.00	0.00	0.00	0.00
**md**	0.00	0.00	0.00	0.00	**0.87**	0.00	0.00	0.00	0.00	0.00
**se**	**0.33**	0.00	0.00	0.00	**0.33**	0.00	0.00	0.00	**0.33**	0.00
**sm**	**1.00**	0.00	0.00	0.00	0.00	0.00	0.00	0.00	0.00	0.00
**tm**	**0.17**	**0.17**	0.00	**0.17**	0.00	0.00	**0.42**	0.00	0.00	0.00
**tp**	0.00	0.00	0.00	0.00	**0.57**	0.00	0.00	0.00	0.00	**0.23**

Table W. Transition Frequencies matrix (lag -1) for DE grooming technique with probability higher than 15%

	em	fs	gl	hp	hs	md	se	sm	tm	tp
**em**	0	0	0	0	0	0	**0.22**	0	0	0
**fs**	0	**0.33**	0	0	0	0	0	0	0	0
**gl**	0	0	**0.24**	0	0	0	0	0	0	0
**hp**	0	**0.33**	0	0	**0.17**	0	0	0	0	0
**hs**	0	0	**0.62**	**0.78**	**0.71**	**0.83**	0	**0.5**	0	**0.67**
**md**	0	0	0	0	0	0	0	0	0	0
**se**	**0.43**	0	0	0	0	0	0	0	**0.25**	0
**sm**	**0.29**	0	0	0	0	0	0	0	0	0
**tm**	**0.29**	**0.33**	0	0	0	0	**0.56**	0	0	0
**tp**	0	0	0	0	0	**0.17**	**0.22**	**0.5**	**0.75**	**0.23**

Table X. Final Transition Frequencies matrix for DE grooming technique. There are 30 relevant transitions. Bold values are higher than the average (62%)

	em	fs	gl	hp	hs	md	se	sm	tm	tp
**em**	0	0	0	0.57	0	0	0.51	0	0	0
**fs**	0	**0.73**	0	0.4	0	0	0	0	0	0.2
**gl**	0	0	0.48	0	**0.76**	0	0	0	0	0
**hp**	0	0.33	0	0	**0.96**	0	0	0	0	0
**hs**	0	0	**0.62**	**0.95**	**1.42**	**0.83**	0	0.5	0	**0.67**
**md**	0	0	0	0	**0.87**	0	0	0	0	0
**se**	**0.76**	0	0	0	0.33	0	0	0	0.58	0
**sm**	**1.29**	0	0	0	0	0	0	0	0	0
**tm**	0.45	0.5	0	0.17	0	0	**0.97**	0	0	0
**tp**	0	0	0	0	0.57	0.17	0.22	0.5	**0.75**	0.46

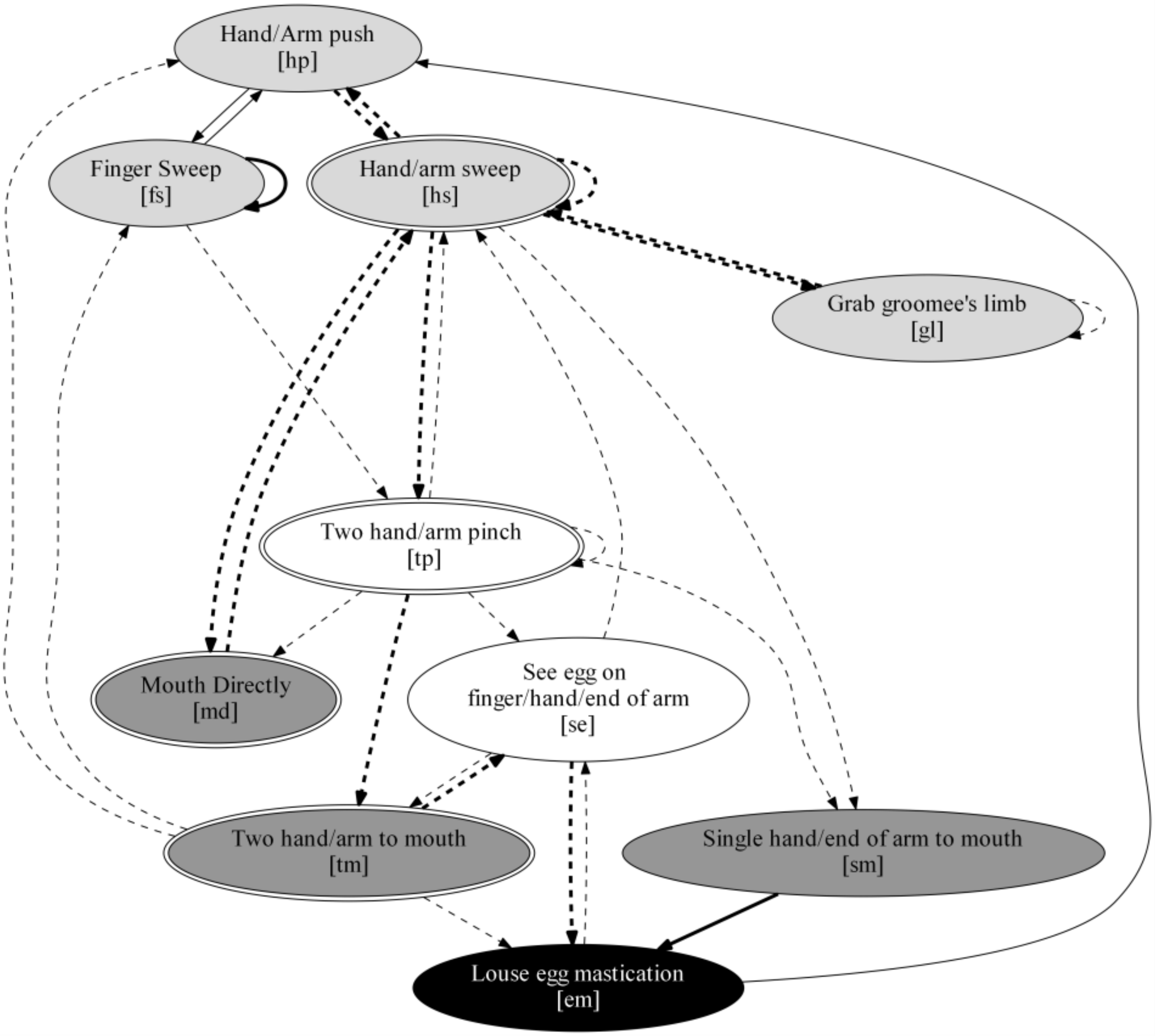

Figure G. Disabled grooming technique DE

The process begins at the top of the figure. Node color matches with the four grooming stages described in Fig 4. Solid lines represent transitions common with the ND technique. Dashed lines show transitions not found in the ND technique. Transitions with probabilities higher than 62% are indicated with thicker lines. Double-bordered movements (4) are performed only by the disabled individuals that use this technique.

Table Y. Joint Frequencies matrix for DF grooming technique

	Target:				
**Given:**	**em**	**ep**	**hs**	**md**	**Totals**
**em**	0	0	5	0	5
**ep**	0	10	137	6	153
**hs**	0	125	1065	111	1301
**md**	5	19	91	7	122
**Totals**	5	154	1298	124	1581

Table Z. Transition Frequencies matrix (lag 1) for DF grooming technique with probability higher than 15%

	**em**	**ep**	**hs**	**md**
**em**	0.00	0.00	**1.00**	0.00
**ep**	0.00	0.00	**0.90**	0.00
**hs**	0.00	0.00	**0.82**	0.00
**md**	0.00	**0.16**	**0.75**	0.00
**hs**	0.00	0.00	**0.82**	0.00

Table AA. Transition Frequencies matrix (lag -1) for DF grooming technique with probability higher than 15%

	**em**	**ep**	**hs**	**md**
**em**	0.00	0.00	0.00	0.00
**ep**	0.00	0.00	0.00	0.00
**hs**	0.00	**0.81**	**0.82**	**0.90**
**md**	**1.00**	0.00	0.00	0.00

Table AB. Final Transition Frequencies matrix for DF grooming technique. There are 8 relevant transitions. Bold values are higher than the average (90%)

	**em**	**ep**	**hs**	**md**
**em**	0.00	0.00	**1.00**	0.00
**ep**	0.00	0.00	**0.90**	0.00
**hs**	0.00	0.81	**1.64**	**0.90**
**md**	**1.00**	0.16	0.75	0.00

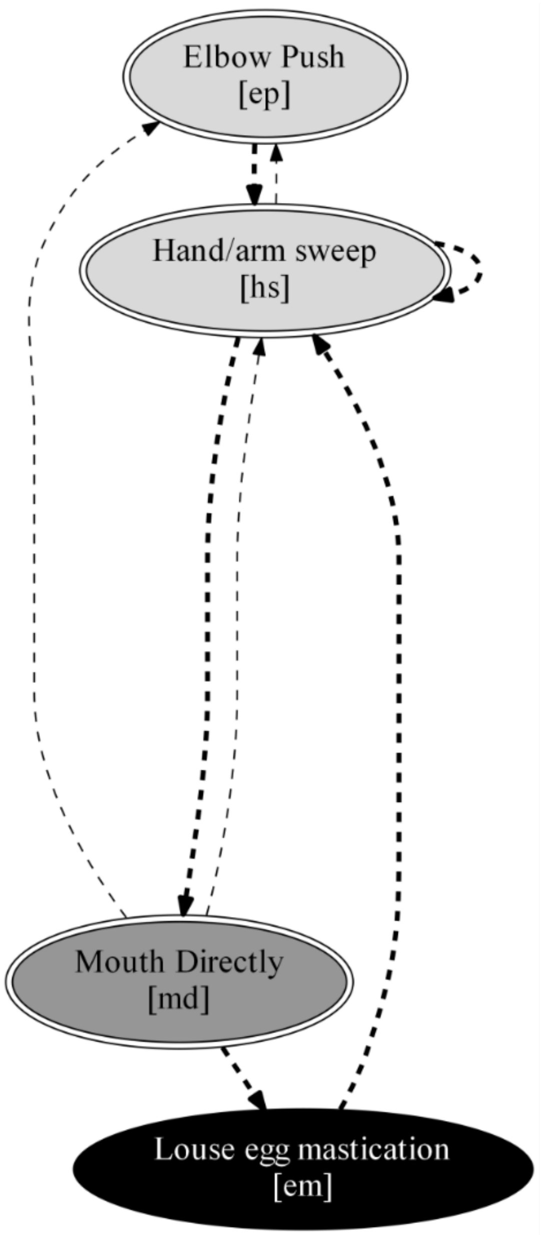

Figure H. Disabled grooming technique DF

The process begins at the top of the figure. Node color matches with the four grooming stages described in Fig 4. Solid lines represent transitions common with the ND technique. Dashed lines show transitions not found in the ND technique. Transitions with probabilities higher than 90% are indicated with thicker lines. Double-bordered movements (3) are performed only by the disabled individuals that use this technique.
