# Supplementary material for: Social grooming efficiency and techniques are influenced by manual impairment in free-ranging Japanese macaques (Macaca fuscata)
Source: PLoS One. 2020 Feb 21;15(2):e0228978. doi: 10.1371/journal.pone.0228978 (PMC7034802; doi:10.1371/journal.pone.0228978)
Supplement: S4 File — (PDF) [file pone.0228978.s004.pdf]

Table A. Grooming efficiency values for each disabled and nondisabled technique

Grooming technique	Grooming efficiency			
	Removal efficiency		Movement efficiency	
	N	Average	N	Average
ND	58	5.7789	57	38.7368
NDM	50	4.66	50	46.2023
DA	33	4.5455	33	50.4400
DB	13	3.3154	12	66.7125
DC	27	1.6296	20	77.7083
DD	12	1.3333	8	68.2917
DE	12	0.5833	5	85.6667
DF	11	0.4545	3	82.6667

Table B. Results of Mann-Whitney test examining differences in grooming efficiency between Nondisabled grooming techniques ND and NDM

	Grooming technique	W	p-value
Removal efficiency (number of eggs per 2 mins)	ND	1680	0.155
	NDM		
Movement efficiency (number of movements per egg removed)	ND	1098.5	**0.04179**
	NDM		

Table C. Results of Mann-Whitney test examining differences in Grooming efficiency between Disabled and Nondisabled grooming techniques

Grooming efficiency	**Nondisabled grooming technique**	**Disabled grooming technique**	**W**	**p-value**
Removal efficiency (number of eggs per 2 mins)	ND	DA	861	0.7388
		DB	259	0.07907
		DC	220	**8.968e-08**
		DD	80	**2.757e-05**
		DE	33.5	**8.787e-07**
		DF	27	**1.576e-06**
	NDM	DA	824.5	0.2726
		DB	275.5	0.401
		DC	232	**1.797e-06**
		DD	82	**9.297e-05**
		DE	28	**1.09e-06**
		DF	23	**1.997e-06**
Movement efficiency (number of movements per egg removed)	ND	DA	1240	**0.01229**
		DB	498	**0.01382**
		DC	969.5	**3.563e-06**
		DD	362	**0.007677**
		DE	246	**0.007744**
		DF	144	**0.04913**
	NDM	DA	879	0.6186
		DB	381.5	0.1489
		DC	785	**0.0002162**
		DD	291	**0.04125**
		DE	207	**0.01702**
		DF	119	0.09402
