# Supplementary material for: Social grooming efficiency and techniques are influenced by manual impairment in free-ranging Japanese macaques (Macaca fuscata)
Source: PLoS One. 2020 Feb 21;15(2):e0228978. doi: 10.1371/journal.pone.0228978 (PMC7034802; doi:10.1371/journal.pone.0228978)
Supplement: Attachment — Submitted filename: Plos One Review.docx [file pone.0228978.s006.docx]

I enjoyed reading this paper and appreciated the authors clarity with respect to overall organization, writing, and analysis. The authors provide an interesting contribution to the literature that focuses on behavioral flexibility and primates’ adaptation to disability. I suggest it be accepted for publication in *Plos One*. I proposed below a few recommendations for the authors to consider in any revision that may occur.

Minor editorial suggestions:

- Line 41 should the world “in” be “any”?
- Lines 52 and 70 comma needed after e.g.? (you used commas after i.e.)
- Line 80 macaque (lower case)
- Line 152 order; however,
- Line 163 samples (lower case)
- Some inconsistencies in case for your ethogram behaviors. It appears that you are following the pattern of capitalizing the first word and lower case for the rest. With that in mind, please see lines 288, 316-319, 331, 341, 375, 388, 398, 399, 402, 415, 455, 487, 488, 498
- Line 513 efficiency; however,
- Line 546 I suggest you delete the word “properly”
- Line 562 conditions (plural)
- Lines 561-566 The information might read more clearly broken into two sentences, for example, at line 564 grooming. However,

Analytical suggestions:

N.B.: In my comments below, D=Disabled individuals, ND=Nondisabled individuals

- Lines 542-544 some additional discussion of this might be helpful. It occurs to me that one response that a potential groomee might have to being approached for grooming by a D (and therefore less efficient) individual would be to refuse the invitation to groom. The source cited on lines 544-5 (14) may address this, but it was unclear to me whether you meant D individuals being invited to groom others, or D individuals being groomed. I am wondering if D individuals groom more frequently with relatives (see also below). I wondered whether D females had fewer grooming partners compared to ND females…I apologize if you reported this, and I missed it! But I couldn’t easily find it on my first or second read. I suspect that is the case and thought it might relate to your discussion of reduced efficiency by ND females.
- An interesting characteristic of this population is that you have information on maternal relatedness. As I read your paper, I wondered how matrilineal relationships might impact on your data and/or on your interpretations of it. Lines 82 -87 raise the issue of technique being transmitted through kin lines. Are you arguing in the following sentences that trial and error learning overrides this? I felt like this could be discussed more thoroughly at lines 464-467 and specifically wondered whether your six ND females who used the *mouth directly* to groom were related to any of the D females. It would be helpful to indicate kin structure in S1 table, as I (again) wondered about the relationships among the females you studied. Lines 440-454 is another point in the manuscript where I had questions about kinship and how matriline membership might be (or might not be!) patterning the data. If possible, I think your arguments would be strengthened by including matriline as a variable in your analysis. It might also be helpful to indicate whether disabilities in this population are clustered within matrilines, or are disabilities distributed across the population?
- Another compelling feature of your population is that you likely have more individuals living to be old than is true of unprovisioned populations. I did not see age reported in S1 table, and I suggest that be included. The disability classifications you used in this paper focused on those that influence manual dexterity/grip, but it occurred to me that visual impairment might be occurring in your older females (similar to that reported for bonobos by Ryu, Graham, Sakamaki, and Furuichi Current Biology 26, R1119–R1136). As was true for matriline membership, I wondered whether age explained any of the patterning in your dataset. If your ND mouth directly groomers are old females, that might account for their use of that technique. It seems that relying more on one’s mouth might occur as the ability to see the louse is reduced or lost.

Thank you for this opportunity to review your interesting work.
